# Serum biomarker profiles and response to neoadjuvant chemotherapy for locally advanced breast cancer

**DOI:** 10.1186/bcr2096

**Published:** 2008-05-12

**Authors:** Brian M Nolen, Jeffrey R Marks, Shlomo Ta'san, Alex Rand, The Minh Luong, Yun Wang, Kimberly Blackwell, Anna E Lokshin

**Affiliations:** 1 University of Pittsburgh Cancer Institute, Hillman Cancer Center, Suite 1.19d, 5117 Centre Avenue, Pittsburgh, PA 15213, USA; 2 Department of Surgery, Duke University Medical Center, Box 3873 Med Ctr Durham, NC 27710, USA; 3 Department of Mathematical Sciences, Carnegie Mellon University, Wean Hall, Room 6113, Pittsburgh, PA 15213-3890, USA; 4 Department of Biostatistics, Graduate School of Public Health, University of Pittsburgh, 130 Desoto Street, 311 Parran Hall, Pittsburgh, PA 15261, USA; 5 Department of Radiation Oncology, Duke University Medical Center, Box 3893 Med Ctr Durham, NC 27710, USA; 6 Department of Medicine, Duke University Medical Center, Box 3893 Med Ctr Durham, NC 27710, USA; 7 Department of Medicine, School of Medicine, University of Pittsburgh, 1218 Scaife Hall 3550 Terrace Street, Pittsburgh, PA 15213, USA; 8 Department of Pathology, University of Pittsburgh, S-417 BST, 200 Lothrop Street, Pittsburgh, PA 15261, USA; 9 Department of Obstetrics and Gynecology RS, University of Pittsburgh, 300 Halket Street Pittsburgh, PA 15213, USA

## Abstract

**Introduction:**

Neoadjuvant chemotherapy has become the standard of care for the diverse population of women diagnosed with locally advanced breast cancer. Serum biomarker levels are increasingly being investigated for their ability to predict therapy response and aid in the development of individualized treatment regimens. Multianalyte profiles may offer greater predictive power for neoadjuvant treatment response than the individual biomarkers currently in use.

**Methods:**

Serum samples were collected from 44 patients enrolled in a phase I–II, open-label study of liposomal doxorubicin and paclitaxel in combination with whole breast hyperthermia for the neoadjuvant treatment of locally advanced breast cancer (stage IIB or stage III). Samples were collected prior to each of four rounds of treatment and prior to definitive surgery. Samples were assayed by Luminex assay for 55 serum biomarkers, including cancer antigens, growth/angiogenic factors, apoptosis-related molecules, metastasis-related molecules, adhesion molecules, adipokines, cytokines, chemokines, hormones, and other proteins.

**Results:**

Biomarker levels were compared retrospectively with clinical and pathologic treatment responses. Univariate analysis of the data identified several groups of biomarkers that differed significantly among treatment outcome groups early in the course of neoadjuvant chemotherapy. Multivariate statistical analysis revealed multibiomarker panels that could differentiate between treatment response groups with high sensitivity and specificity.

**Conclusion:**

We demonstrate here that serum biomarker profiles may offer predictive power concerning treatment response and outcome in the neoadjuvant setting. The continued development of these findings will be of considerable clinical utility in the design of treatment regimens for individual breast cancer patients.

**Trial registration:**

#NCT00346229.

## Introduction

Locally advanced breast cancer (LABC) is a generalized diagnosis that includes all stage III disease and a subset of stage IIB disease [1]. The clinical definition of LABC continues to evolve and differ among physicians, and now includes nonmetastatic T3 or T4 tumors as well as N2/N3 disease involving limited metastasis [2], thus broadening the already diverse spectrum of LABC presentations. According to the American College of Surgeons Data Base, approximately 6% of all US breast cancer cases present as stage III [3]. This number has declined dramatically over the past decade due to improved screening and detection practices. The 5-year relative survival rate for stage III breast cancer is approximately 50%, compared with 87% for stage I. The median survival for women with stage III disease is 4.9 years [1].

Neoadjuvant chemotherapy (NAC), the delivery of systemic chemotherapy prior to surgical resection, has emerged as the preferred initial component of therapy for patients diagnosed with LABC in an effort to enhance the prospect of breast-conserving surgery and to render inoperable tumors resectable [4,5]. NAC offers the theoretical advantages of early initiation of systemic therapy, delivery of drugs through intact vasculature, *in vivo *assessment of therapy response, and the opportunity to study the biological effects of chemotherapy [6]. Preoperative systemic chemotherapy may also eradicate distant micrometastases and thus improve the overall effectiveness of treatment [7]. Several studies comparing NAC with more traditional adjuvant chemotherapy have found similar survival rates between the two options [5,8,9], and the use of NAC is further supported by findings that delaying surgery for the administration of chemotherapy does not adversely affect treatment outcome when compared with adjuvant chemotherapy [10,11].

A pathologic complete response (pCR) following NAC implies the absence of residual invasive or *in situ *disease and correlates strongly with both prolonged disease-free survival and overall survival [12,13]. A recent review of several randomized clinical trials of NAC for operable breast cancer reported a response rate of 49% to 94% with a pCR rate of 4% to 34% [12]. In patients treated with NAC, 60% to 80% demonstrate some clinical response with 10% to 20% achieving a clinical complete response (cCR) [4]. Clinical response, however, often does not correlate with pathologic response as a full one-third of patients achieving a cCR are found to have pathologic evidence of residual disease [9,14]. Despite these difficulties in assessing response, patients demonstrating complete clinical or pathologic responses to NAC generally achieve improved outcomes to overall treatment [14,15].

Accurate modalities for assessing chemotherapy response are critical to the evaluation and expansion of the use of NAC for breast cancer. Conventional methods including clinical examination, mammogram, and breast ultrasound are incorrect in identifying pCR patients in nearly one-half of all cases [2]. Several groups have reported promising results in their attempts to predict treatment response utilizing unconventional techniques such as diffuse optical spectroscopy and magnetic resonance imaging [16,17]. A growing number of investigators have begun to utilize the preoperative nature of NAC to conduct intreatment analyses of molecular markers that may predict response to therapy. The emergence of new technologies such as transcriptional and proteomic profiling has greatly aided such investigations [18,19]. For instance, it has been reported that mutations in p53 are associated with a lower response rate following NAC [20,21], while coexpression of HER-2/Neu and topoisomerse II is associated with greater response rates [22]. Measurements of the traditional breast cancer markers CA15-3 and HER-2/Neu, however, have demonstrated only limited predictive value in the NAC setting [23,24].

In the present study we examine a diverse panel of serum biomarkers in order to identify individual biomarkers and combinations that may be useful in predicting treatment response early in the course of NAC for the treatment of LABC.

## Materials and methods

### Patients

Serum samples were collected from patients enrolled in a phase I–II, open-label study of liposomal doxorubicin (Evacet; Elan Corp., Stevenage, UK) and paclitaxel (Bristol Myers Squibb, Princeton, NJ, USA) in combination with whole breast hyperthermia for the neoadjuvant treatment of LABC (stage IIB or stage III). This trial required informed consent and was conducted under the approval of the Duke University Institutional Review Board. Protocol-eligible patients were treated with the combination of Evacet, paclitaxel, and hyperthermia every 3 weeks. The hyperthermia procedure has been described previously [25].

Following neoadjuvant therapy, patients received appropriate surgical removal of their primary breast tumor as well as axillary lymph node dissection. Immediately after surgery, patients underwent radiation therapy followed by an additional eight cycles each of 21-day standard dose cyclophosphamide (600 mg/m^2^), methotrexate (40 mg/m^2^), 5-fluorouracil (600 mg/m^2^) and appropriate hormonal therapy.

The clinical trial accrued a total of 47 patients. Three patients were deemed nonevaluable because of failure to complete all four cycles of the neoadjuvant portion of the trial. The clinical characteristics of the patient study group are presented in Table 1.

**Table 1:** Clinical characteristics of study patients

Characteristic	Number of patients
Patients in study	
Total enrolled	47
Total completing treatment	44
Clinical stage pretreatment	
Stage IIB	15
Stage IIIA	12
Stage IIIB	16
Clinical response	
Complete	12
Partial	20
Stable disease	12
Pathologic response	
Complete	4
Partial	23
Stable disease	17

#### Collection and storage of blood serum

Serum samples were obtained prior to the start of neoadjuvant therapy (pretreatment), prior to cycles 2, 3, and 4 of neoadjuvant therapy, and prior to definitive surgery. Blood was drawn using standard phlebotomy procedures and was collected without anticoagulant. Blood was allowed to coagulate for up to 2 hours at room temperature. Sera were separated by centrifugation, immediately aliquoted, frozen, and stored at -80°C. No more than two freeze–thaw cycles were allowed for any sample.

### Multiplexed bead-based immunoassay

The xMAP™ bead-based technology (Luminex Corp., Austin, TX, USA) permits simultaneous analysis of numerous analytes in a single sample. Fifty-five bead-based xMAP™ immunoassays for a variety of serum biomarkers were utilized in the present study (Table 2).

**Table 2:** Complete list of biomarkers tested

Biomarker category	Individual biomarkers
Cancer antigens/oncogenes	α-Fetoprotein, CA 125, CA 19-9, CA 15-3, CA 72-4, carcinoembryonic antigen
Cytokines/chemokines/receptors	Eotaxin, fractalkine, granulocyte–macrophage colony-stimulating factor, IFNγ, IL-10, IL-12p70, IL-13, IL-1β, IL-1Rα, IL-2, IL-2R, IL-4, IL-5, IL-6, IL-6R, IL-7, IL-8, IP-10, migration inhibitory factor, MIP-1β, soluble CD40L, TNFα, TNF-R1, TNF-R2
Growth/angiogenic factors	Epidermal growth factor receptor, ErbB2, insulin-like growth factor binding protein 1, transforming growth factor alpha
Proteases	Kallikrein 10, MMP-2, MMP-3, MMP-9
Hormones	Adrenocorticotropic hormone, follicle-stimulating hormone, growth hormone, luteinizing hormone, prolactin, thyroid-stimulating hormone
Adipokines	Adiponectin
Apoptosis-related molecules	Cyfra 21-1, DR5, soluble Fas, soluble Fas ligand
Metastasis-related molecules	Myeloperoxidase, tissue plasminogen activator inhibitor 1
Adhesion molecules	Soluble intracellular adhesion molecule 1, soluble vascular cell adhesion molecule 1
Other proteins	HE4, mesothelin

Assays for ErbB2, epidermal growth factor receptor (EGFR), CA 15-3, carcinoembryonic antigen, Cyfra 21-1, CA 19-9, CA 72-4, α-fetoprotein, mesothelin, insulin-like growth factor binding protein 1, human kallikrein 10, and HE4 were developed in the UPCI Luminex Core Facility [26]. The inter-assay variability of each assay was 5% to 11%, and the intra-assay variability was 2% to 9%. Assays for MMP-2 and MMP-3 were obtained from R&D Systems (Minneapolis, MN, USA), assays for MIP-1β, eotaxin, IP-10, IL-2R, IL-1Rα, IL-6R, DR5, TNF-RI, and TNF-RII were obtained from Invitrogen (Camarillo, CA), and the remaining assays were obtained from Millipore/Linco Research (St Charles, MO, USA). Overall, eight different multiplexed panels were used.

### Multiplex analysis

Assays were performed according to the manufacturers' protocols. Luminex Core Facility assays were performed as described previously [27]. Samples were analyzed using the Bio-Plex suspension array system (Bio-Rad Laboratories, Hercules, CA, USA). Biomarker expression levels were expressed as median fluorescent intensities generated by analyzing 50 to 100 microbeads for each analyte in each sample. The concentrations of analytes were quantitated from median fluorescence intensities using standard curves generated by Bio-Rad five-parameter curve fitting) to the series of known concentrations for each analyte.

### Statistical analysis

#### Clinical response

The Mann–Whitney nonparametric *t *test was used to evaluate the significance of differences in serum biomarker levels expressed as the median fluorescence intensity between treatment response groups separated by treatment timepoints. The level of significance was taken as *P *< 0.05. For multivariate analysis of biomarker combinations, a CART classification tree [28-30] diagnostic model was created. The Statistical Analysis System (SAS version 9: SAS Institute, Inc., Cary, NC, USA) was used to fit the logistic regressions using PROC LOGISTIC. The best subset for each size panel of analytes was identified through the brand and bound algorithm of Furnival and Wilson [31]. This algorithm maximizes the score function over all possible combinations of analytes for any given size panel. The Statistical Analysis System was also used to fit the logistic regressions and to identify the best subsets for each size panel of biomarkers. Panels were generated from size 1 to size 10.

Sensitivities were estimated for specificities of 90%, 95%, and 98% by ranking the predicted fit for each control subject, determining the cutoff points corresponding to these levels of specificity, and applying the cutoff points to the ranked predictions for the alternative treatment response group. To minimize overfitting bias, leave-one-out cross-validation was used. The MATLAB routines *treefit *and *treeval *were used. For panel selection, markers were selected incrementally. Given an existing subset of the markers, each marker was considered a potential addition to the panel. We began with no markers and added until little additional progress was made.

#### Pathologic response

Within each timepoint of treatment, biomarker expression values – expressed as the median fluorescence intensity – were adjusted by the following procedure. Quantile normalization was performed using the normalize BetweenArrays function (limma package) in R [32], missing values were filled in using k-nearest neighbor imputation, and values were log-transformed.

Normalized values were filtered according to a univariate, two-sided *t *test. From this filtered set, values were progressively included and excluded from a stepwise regression model. The final logistic regression model was then subjected to leave-one-out cross-validation to assess the predictive value.

## Results

### Multiplex analysis of serum levels of various biomarkers in LABC patients receiving neoadjuvant chemotherapy

Of the 44 evaluable patients enrolled in the clinical trial, 12 demonstrated a cCR, 20 demonstrated a clinical partial response, and 12 had no response. Of the same group, four patients demonstrated a pCR, 23 demonstrated a pathologic partial response, and 17 had no response. One patient out of the total 44 did not have blood drawn prior to the second cycle of NAC and was excluded from the analysis for that timepoint.

A bead-based 55-biomarker panel was utilized to screen the sera from patients. The biomarkers included cancer antigens, growth/angiogenic factors, apoptosis-related molecules, metastasis-related molecules, adhesion molecules, adipokines, cytokines, chemokines, hormones, proteases, and other proteins (Table 2). Biomarker levels were compared at each treatment timepoint between complete responders, partial responders, and nonresponders within each response classification (clinical or pathologic).

Combinations of biomarkers were evaluated by multivariate analysis for the ability to predict a particular response. Our analysis was limited to serum samples collected prior to the initiation of NAC and prior to the second cycle of therapy. We did not identify any significant correlations between biomarker levels and pathologic response at the pretreatment timepoint.

### Analysis of pretreatment serum biomarker levels according to clinical response

**Figure 1:**
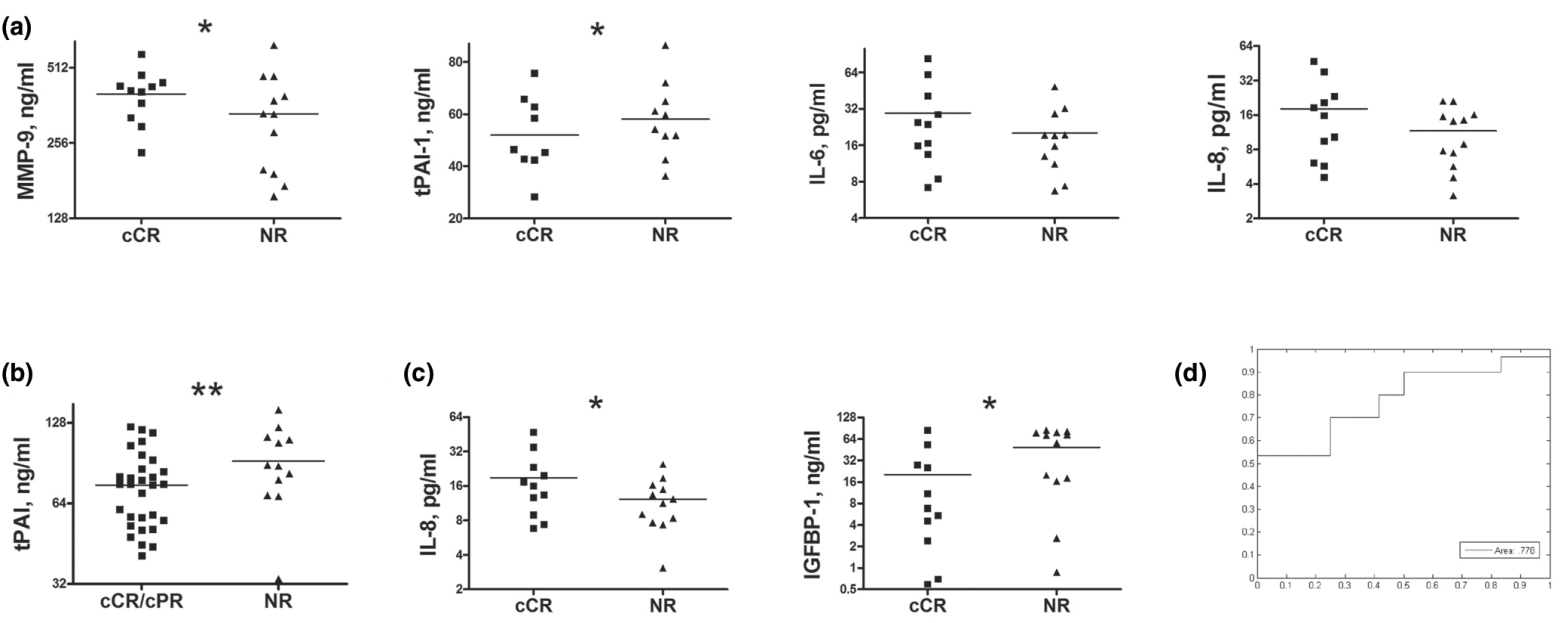
Serum biomarker analysis of neoadjuvant chemotherapy for locally advanced breast cancer according to clinical response. **(a) **Pretreatment serum levels of IL-6, IL-8, MMP-9, and tissue plasminogen activator inhibitor 1 (tPAI-1) were compared between 11 patients achieving a clinical complete response (cCR) and 12 patients demonstrating no response (NR) to neoadjuvant chemotherapy (NAC). **(b) **Pretreatment serum levels of tPAI-1 were compared between 30 patients achieving a cCR or clinical partial response (cPR) and 12 patients demonstrating NR to NAC. **(c) **Serum levels of insulin-like growth factor binding protein 1 (IGFBP-1) and IL-8 measured prior to the second round of NAC were compared between 11 patients achieving a cCR and 11 patients demonstrating NR to NAC. **(d) **Cumulative receiver operating characteristics for responders versus nonresponders based on pretreatment serum levels of tPAI-1. Statistical significance: *P < 0.05; **P < 0.01.

For this analysis, 11 patients achieving a cCR were compared with 12 patients demonstrating no response. Serum levels of MMP-9 were significantly higher in patients achieving a cCR while levels of tissue plasminogen activator inhibitor 1 (tPAI-1) were significantly lower in the same group (*P *< 0.05, Figure 1a). Higher levels of IL-6 and IL-8 were also observed in patients achieving a cCR, although this observation was not statistically significant (*P *< 0.07, Figure 1).

A CART classification tree analysis of serum biomarker levels from these patients identified a three-biomarker panel consisting of α-fetoprotein, soluble vascular cell adhesion molecule 1, and MMP-9 that could distinguish between the response groups with 83% sensitivity and 91% specificity (Table 3). Interestingly, when the 11 patients achieving a cCR were combined with 19 patients achieving a clinical partial response and were compared with the nonresponders, tPAI-1 alone could distinguish responders from nonresponders with 75% sensitivity and 77% specificity (Table 3 and Figure 1d). Serum levels of tPAI-1 were significantly lower in responders (cCR and clinical partial response) compared with nonresponders (*P *< 0.007, Figure 1b).

**Table 3:** Predictive power of multimarker panels

Panel	Timepoint	Response type	Sensitivity (%)	Specificity (%)
α-Fetoprotein, soluble vascular cell adhesion molecule 1, MMP-9	Pretreatment	Clinical complete response versus no response	83	91
Tissue plasminogen activator inhibitor 1	Pretreatment	Clinical complete response/clinical partial response versus no response	75	77
MMP-3, luteinizing hormone, thyroid-stimulating hormone	Pre-cycle 2	Clinical complete response versus no response	82	73
ErbB2, epidermal growth factor receptor, migration inhibitory factor, MMP-2, CD40 ligand	Pre-cycle 2	Pathologic complete response/pathologic partial response versus no response	85	69

### Analysis of serum biomarker levels according to clinical response prior to the second cycle of neoadjuvant chemotherapy

For this analysis, 11 patients achieving a cCR were compared with 11 patients demonstrated no response. Serum levels of IL-8 were significantly higher and those of insulin-like growth factor binding protein 1 were significantly lower in patients achieving a cCR (*P *< 0.05, Figure 1c). CART classification tree analysis of serum biomarker levels from these patients identified a three-biomarker panel consisting of MMP-3, luteinizing hormone, and thyroid stimulating hormone that could distinguish between clinical response groups with 82% sensitivity and 73% specificity (Table 3).

### Analysis of serum biomarker levels according to pathologic response prior to the second cycle of neoadjuvant chemotherapy

**Figure 2:**
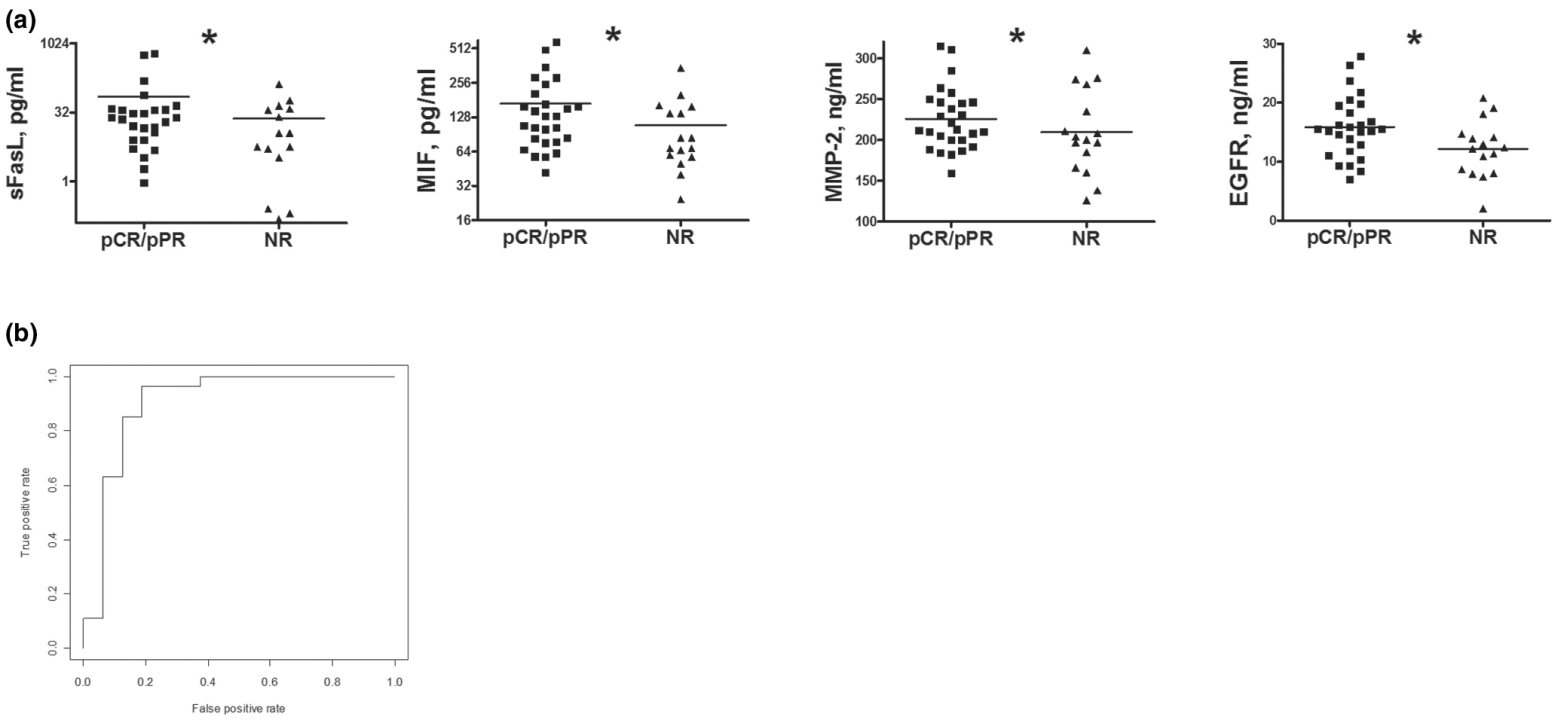
Serum biomarker analysis of neoadjuvant chemotherapy for locally advanced breast cancer according to pathologic response. **(a) **Serum levels of epidermal growth factor receptor (EGFR), soluble Fas ligand (sFasL), migration inhibitory factor (MIF), and MMP-2 measured prior to the second round of neoadjuvant chemotherapy (NAC) in 27 patients achieving a pathologic complete response (pCR) or partial pathologic response (pPR) and 16 patients demonstrating no response (NR) to NAC. **(b) **Cumulative receiver operating characteristics for patients achieving a pCR or pPR versus nonresponders to NAC based on serum levels of ErbB2, EGFR, MIF, MMP-2, and CD40 measured prior to the second cycle of NAC. Statistical significance: *P < 0.05.

For this analysis, 27 patients achieving a pCR or pathologic partial response were compared with 16 patients demonstrating no response. Serum levels of EGFR, soluble Fas ligand, migration inhibitory factor (MIF), and MMP-2 were significantly higher in responders compared with non-responders (*P *< 0.05, Figure 2a).

A logistic regression analysis of the serum biomarker levels in these patients identified a five-biomarker panel consisting of ErbB2, EGFR, MIF, MMP-2, and CD40L that could distinguish responders from nonresponders with 85% sensitivity and 69% specificity (Table 3 and Figure 2b).

## Discussion

The heterogeneity displayed by patients diagnosed with LABC runs counter to the rationale for generalized treatment regimens. A wide array of treatment options exist for the treatment of breast cancer – including adjuvant and NAC, hormone therapy, radiotherapy, and surgery – and the vast majority of these options have been well researched. The ability to dynamically tailor the components of a particular treatment regimen on a patient by patient basis would be invaluable. Such an accomplishment will require the identification and development of improved prognostic factors on which to base therapeutic decisions, since currently used measurements of clinical and radiological response lack the necessary precision. In the present article we demonstrate the predictive value of serum biomarkers for the response to NAC for LABC. The diverse nature of the biomarker relationships identified in the study underscores the diversity of the disease characteristics present in the patient population.

Previous studies have examined the value of biomarkers such as carcinoembryonic antigen, CA 15-3, MMP-2, MMP-9, tissue polypeptide antigen (TPA), tissue polypeptide-specific antigen (TPS), EGFR, and HER-2/neu in predicting response to NAC for breast cancer [33-36]. The results of these investigations have been mixed. To our knowledge, the panel of serum biomarkers examined in the present study is the largest and most diverse to date and the majority of the relationships we identify have not been described previously.

Our results indicate that elevated serum levels of IL-6, IL-8, and MMP-9 prior to the initiation of treatment correlate with improved clinical response. The observed increases in IL-6 and IL-8 serum levels of 2.74 and 3.13 pg/ml, respectively (Figure 1a), were not significant in our study given the limited 23-patient enrollment. Utilizing these data, we predict that an enrollment of 59 patients would be sufficient to confer significance (*P *< 0.05) upon these observations with a power of 0.9. Increased serum levels of the inflammatory cytokines IL-6 and IL-8 have been associated with poor prognosis in women with breast cancer [37,38]. These cytokines have also been implicated in the blood-borne response to paclitaxel treatment [39]. The prognostic value of MMP-9 serum levels is currently unclear [33], but several studies have examined extensively the role of matrix metalloproteinases in breast cancer [40,41].

Our results also demonstrate a correlation between lower pretreatment serum levels of tPAI-1 and improved clinical response. In fact, tPAI-1 levels alone were able to discriminate responders from nonresponders with 75% sensitivity and 77% specificity prior to the start of NAC. tPAI-1 is a major physiological inhibitor of tissue-type plasminogen activators and has been implicated in tumor growth, invasion, and angiogenesis. The function of tPAI-1 as a regulator of plasminogen activation places it in a position to modulate degradation of the extracellular matrix and antiangiogenic effects mediated by plasmin and angiostatin, respectively. Several groups have illustrated experimentally the role of tPAI-1 in tumor progression and its negative prognostic value in breast cancer [42-44].

In sera collected after patients had received the initial cycle of chemotherapy we observed that increased levels of IL-4, IL-8, and adrenocorticotropic hormone and decreased levels of insulin-like growth factor binding protein 1 all correlated with improved clinical response at differing levels of significance. The increase in levels of inflammatory cytokines following chemotherapy is in line with clinical expectations and is evidence that the therapeutic agents are active in the patient's system. The roles of the insulin-like growth factor system and hormones in the response to chemotherapy have not been significantly evaluated; however, both groups have known roles in the development of breast cancer [45,46].

With regards to pathologic response, we observed a correlation between increased levels of soluble Fas ligand, MMP-2, MIF, and EGFR and improved response. A proapoptotic response following chemotherapy, as evidenced by increased soluble Fas ligand in the sera, is to be expected. Our observation of increased levels of MMP-2 in the sera of responders supports the notion of matrix metalloproteinase involvement in breast cancer. The precise role of macrophage MIF in breast cancer development and treatment response remains unknown, but MIF has been implicated in tumor cell survival pathways [47]. The value of EGFR serum and tissue levels in predicting response to chemotherapy has been examined previously with inconclusive results [43,48]. Increased expression of EGFR has been demonstrated elsewhere to suggest a poor prognosis in breast cancer [49].

It is noteworthy to mention that our present analysis did not identify any significant relationships between CA 15-3 or HER2/Neu and response to NAC. This observation adds to several sparse and conflicting reports in the literature [23,50,51]. It would appear from this uncertainty that, although these particular biomarkers have shown the most promise in terms of diagnosis and prognosis of breast cancer, they may not offer predictive power for chemotherapy response.

In addition to the individual serum biomarkers found to be significant at each timepoint, our multivariate analysis of the data identified several multimarker panels with predictive value for both clinical response and pathologic response prior to or early in the course of treatment. These multimarker panels demonstrated greater predictive power than any single biomarker and illustrate the potential clinical utility of this type of approach to the design and maintenance of treatment regiments. Previous studies from our group have demonstrated the usefulness of multimarker panels in the early detection of ovarian cancer, endometrial cancer, and head and neck cancers [27,52,53].

## Conclusion

Our relatively small study population limits the predictive power of the panels presented here, but the benefits of a serum biomarker and multimarker approach are clearly illustrated and further studies utilizing larger clinical cohorts, as discussed above, are well warranted. As we increase our knowledge of the biomarkers involved in these panels and continue to identify additional players, our ability to utilize the information we receive from exploratory investigations such as this will increase immensely. Continuing efforts in line with those presented here should bring us closer to providing effective and efficient individualized treatment to women diagnosed with this challenging disease.

## Abbreviations

cCR = clinical complete response; EGFR = epidermal growth factor receptor; IL = interleukin; LABC = locally advanced breast cancer; MIF = migration inhibitory factor; MMP = matrix metalloproteinase; NAC = neoadjuvant chemotherapy; pCR = pathologic complete response; TNF = tumor necrosis factor; tPAI-1 = tissue plasminogen activator inhibitor 1.

## Competing interests

The authors declare that they have no competing interests.

## Authors' contributions

BMN participated in the Luminex assays, oversaw the data analysis, and drafted the manuscript. JRM and KB conceived the study and coordination the transfer of patient samples. ST, AR, TML, and YW carried out the statistical analysis. AEL conceived the study, participated in the study design, and helped to draft the manuscript.
